# Perceptions, social representations, and experiences of suspected Lyme borreliosis among adolescents and their parents in France: A multicenter study

**DOI:** 10.1371/journal.pone.0357185

**Published:** 2026-09-21

**Authors:** Sarah Covasso, Giulia Paoletti, Mathie Lorrot, Sophie Dugué, Stéphanie Cardin, Costanza Puppo, Anaïs Chosidow, Alice Raffetin

**Affiliations:** 1 Department of Infectious Diseases, Tick-Borne Diseases Reference Center, Paris and Northern Region, Intercommunal Hospital of Villeneuve Saint Georges, Villeneuve-Saint-Georges, France; 2 Department of Psychology, Tick-Borne Diseases Reference Center, Paris and Northern Region, Intercommunal Hospital of Villeneuve Saint Georges, Villeneuve-Saint-Georges, France; 3 Department of Pediatrics, University Hospital of Armand Trousseau, Paris, France; 4 Department of Pediatric pain and migraine, University Hospital of Armand Trousseau, Paris, France; 5 Inserm Unity 1290 RESHAPE, University of Lyon 2, France; 6 Department of Pediatrics, Tick-Borne Diseases Reference Center, Paris and Northern Region, Intercommunal Hospital of Villeneuve-Saint-Georges, Villeneuve-Saint-Georges, France; 7 Inserm Unity U955, GEIC2O, Mondor Institute of Medical Research, University Paris-Est Créteil (UPEC), Créteil, France; Universite des Montagnes, CAMEROON

## Abstract

**Objective:**

The variability of Lyme borreliosis (LB) clinical manifestations can complicate diagnosis and lead to prolonged periods of medical uncertainty. There is limited knowledge about how children experience and perceive LB. This study aimed to examine the perceptions, social representations, and experiences of LB among adolescents and their parents.

**Methods:**

Conducted from March to June 2023, this multicenter qualitative study used grounded theory and a dyadic approach with semi-structured interviews to identify themes and develop a theoretical model, adhering to COREQ guidelines. Adolescents and their parents were recruited from pediatric clinics, infectious disease pediatric consultations, and a pediatric pain center, ensuring diverse representations (age, gender, region, and serology results).

**Results:**

We interviewed 19 people. A total of 809 codes were identified and organized into three themes: (1) A personal experience of LB influenced by family history; (2) Ambivalence toward medical discourse; (3) A need for improved knowledge about LB to prevent misinformation. Adolescents expressed a range of emotions, including confusion about the nature of LB, disinterest in the disease itself, and a lack of clarity about their care pathways. They also expressed fear that LB, perceived as “dormant,” could resurface in the future and harm them.

**Discussion:**

The study revealed contrasting experiences of LB between adolescents and their parents, with familial history and external influences shaping adolescents’ and parents’ perceptions, potentially fostering doubts, misunderstandings, and conflicts. Strengthening communication and the role of general practitioners in coordinating with specialized centers is crucial to streamline care pathways and reduce misinformation about LB. **Clinical trials registration number:** NCT05678478; https://clinicaltrials.gov/study/NCT05678478?cond=Lyme%20Borreliosis&term=adolescent&rank=2

## 1. Introduction

Lyme borreliosis (LB) is the most widespread tick-borne disease (TBD) in the northern hemisphere [1,2]. Diagnosis is based on three key factors: exposure to a tick bite, the presence of evocative clinical signs, and, in the case of disseminated forms, a positive serology [3]. This infection primarily affects the skin, joints, and the nervous system [4]. Non-specific, polymorphic, and sometimes disabling subjective symptoms (e.g., fatigue, pain) can occur at all stages of the disease, and may persist for several months even after recommended antibiotic treatment is provided (Post-Treatment Lyme Disease Syndrome, PTLDS) [5]. Treatment involves 14–28 days of antibiotic therapy, depending on the affected area [3,6]. In children, LB most commonly presents as erythema migrans and PTLDS remains rare [7–9].

Most research on LB focuses on its treatment and diagnostic tests, with more emphasis on adults than children. Studies in the social sciences are still limited [10–13], and even more in pediatrics. Nevertheless, the existing literature highlights the importance of considering TBDs, including in children, as legitimate subjects of study in the social sciences. The understanding of LB and its social representations in adults were explored among stakeholders involved in TBDs [12], among patients who had been bitten by ticks and among general practitioners [10]. Several studies in adults have shown that some patients perceived LB as an “invisible risk” [10,14,15], with its management potentially leading to tensions in the doctor-patient relationship [10,16,17]. These types of studies have provided valuable insights into these perspectives and highlighted the need for specific approaches to improve patient care, such as the establishment of multidisciplinary centers for managing TBDs in France and across Europe [10,14,17].

Social representations were initially conceptualised by Moscovici [18–20] and further developed by Jodelet [21] and Laplantine [22]. Social representations are defined as the perceptions, values, and ideas shared by a group, which influence behaviors and social interactions [23]. They enable individuals to interpret and navigate their social environment. These representations are dynamic and evolve with cultural context and lived experiences, offering a fluid perspective on human interactions [23]. They play a structural role in the fields of health and illness as Herzlich demonstrated [24], by shaping how individuals perceive disease and the answers they bring to it. Moreover, in the context of LB, where diagnostic processes and care pathways are often shaped by interactions between patients, their relatives, and healthcare professionals, a dyadic qualitative approach is particularly relevant. The exploration of how experiences, meanings, and decisions are co-constructed through interaction between participants seems important and new. The dyadic approach may also facilitate the identification of convergent and divergent perspectives within the same relational context, providing a more nuanced and ecologically valid understanding of participants’ experiences. By examining these interactions, we aimed to explore how behaviors and perceptions change over time, shaped by the lived experiences of adolescents and their parents. The study also considered the personal and family history of the disease, as well as the care process.

## 2. Methodology

### 2.1. Study design

To explore the perceptions, representations, and experiences of LB among adolescents and their parents, a qualitative approach based on semi-structured interviews using grounded theory [25,26] was chosen. This method was deemed most suitable for understanding their agency, viewpoints, and experiences [25–27]. To examine how representations are developed within the family, considered as a system, a dyadic approach was adopted [28–30]. Family interactions were analyzed in terms of their impact on health behaviors and the representations that emerged. In the parent-child relationship, the reciprocal influence of coping strategies adopted to manage the overall experience of illness can also be identified [30]. The items from the COREQ (Consolidated Criteria for Reporting Qualitative studies) checklist were followed to ensure the quality of the method and analysis [31].

### 2.2. Study population and recruitment

This multicenter study was conducted at the general pediatrics department and at the pain management department of two different university and intercommunal hospitals (March-June 2023). All are involved in the activities of a TBD Reference Center. Teenagers were eligible for inclusion if they were aged ≥12 years and ≤17 years, accompanied by a parent or legal guardian, living in mainland France, and either suspected of having LB, diagnosed with LB, or having had LB in the past. There was no objection to their participation from either the adolescents or their parents. Exclusion criteria included lack of proficiency in spoken French by the adolescents or their parents, or individuals unable to express their consent to participate. Participants were informed about the study during their regular medical appointments. Adolescents were selected to ensure maximum variation in opinions, based on the following criteria: age, gender, exposure to ticks, rural or urban residence, suspected or confirmed LB, and the duration of symptoms (< 6 months or > 6 months). Recruitment continued until no new insights emerged in the interviews (data saturation). As a result, the number of participants was not predetermined.

### 2.3. Survey approach and data collection method

Data were collected through semi-structured individual interviews conducted in person, by phone, or videoconference, following a predefined interview guide [26,31], based on the study’s objectives, relevant literature, and a reflexive approach. The interview guide addressed the debate surrounding LB awareness in France, its impact, and how it influences social perceptions. It also explored how parents’ experiences with LB affect those of the adolescent, as well as the various reactions the questions might elicit from participants. The interview guide was developed around 10 themes detailed in S1 Table. First, it was developed by SC (anthropologist) and GP (psychologist), and pre-tested by CP (researcher in social psychology) and AR (infectious diseases physician). Then it was assessed by pediatricians (ML, AC, SD). Finally, the interview guide was pre-tested with three families to verify the ease of understanding of the questions, their sequence, and to avoid repetition. These exploratory interviews were included in the analysis, as their structure did not differ.

According to the dyadic method [29,32], three interviews were conducted per family (≈1 hour): first, the adolescent, second the parents, and third both. Divergent or conflicting viewpoints between participants were considered analytically valuable rather than problematic. First, interviews were conducted separately to avoid constrained expressions or sensitive points of view. Second, interviews were conducted jointly to capture interactional dynamics, including disagreement, negotiation, and the co-construction of meaning. Participants were encouraged to express their perspectives freely, and no attempt was made to reach consensus during the interviews. When necessary, the interviewer ensured balanced participation to avoid dominance by one individual.

The interviews were recorded on a Dictaphone, if the permission was obtained, with parallel note-taking to ensure accurate transcription of the discussions [31]. At the beginning of each interview, the adolescent was asked whether he/she preferred to be addressed informally or formally [31]. During the interviews, an oral style was maintained, with main questions and follow-up questions. The interviews were then transcribed and pseudonymized. Participants were invited to review the transcripts to ensure the accuracy of their statements. Data collection and analysis occurred in parallel, following an iterative process with constant comparison. The interview guide was revised as analysis of the previous interviews progressed.

### 2.4. Data analysis

The data were recorded in an Excel file secured with an access code. To familiarize with the raw data, a word cloud was created using WordArt (version 4.7.7), answering the question: “*Can you give me three words that come to mind when you think about LB?*” Then, the analysis was conducted based on grounded theory [25,26]: (i) Coding raw information; (ii) Categorizing the identified codes to group them into themes; (iii) Relating these categories to identify the predominant themes; (iv) Connecting the study’s objectives to the findings, further defining the focus of the research; (v) Establishing functional relationships among the results; (vi) Theorizing to reinforce the concepts emerging from the previous stages.

During analysis, discrepancies between participants within each dyad were explicitly examined using a comparative intra-dyadic approach. Rather than reconciling opposing accounts, these differences were coded and interpreted as reflecting distinct experiential, relational, or epistemic positions. We paid particular attention to how disagreements were formulated and managed within the interactions. A latent thematic analysis was conducted to identify underlying meanings and assumptions beyond the explicit content of the exchanges [33,34]. This approach enabled a nuanced understanding of participants’ perspectives while preserving the complexity of relational dynamics.

The analysis was conducted independently by a practicing psychologist (GP) and an anthropologist (SC). This triangulation enabled to deepen the exploration of different dimensions within the results. At the end of the analysis, the findings were shared with the participants to gather their feedback. Translation of the citations was performed by AR who is an English speaker, and then reviewed by a native speaker.

### 2.5. Ethics

The study received approval from the local ethics committee of the Intercommunal Hospital of Créteil, number 2023-03-02. Informed and written consent was obtained from each participant before inclusion in the study, after providing an information sheet and verifying its proper understanding by the adolescents and their family. Patients were recruited between March 2^nd^, 2023 and June 20^th^, 2023.

## 3. Results

Nineteen people (8 families) participated in the study, including three pre-test families. Their sociodemographic characteristics are presented (Table 1). The median age of the adolescents was 13.5 years-old [12,14], ranging from 12 to 17 years-old, and the sex ratio 1.5. Most of the interviewed parents were mothers (n = 7) and there were two fathers. Five adolescents had a confirmed LB. Adolescents were selected to ensure maximum variation in opinions, based on the following criteria: age, gender, exposure to ticks, rural (n = 5) or urban (n = 5) residence, suspected (n = 5) or confirmed LB, and the duration of symptoms (<6 months or >6 months).

**Table 1:** Characteristics of adolescents participating in the study.

Family	Adolescent	Parent interviewed	Proven LB	Recruitment	Interviews	Onset date of the disease
**F1**	**A1**	Father **P1**	No	Consultation	In person	> 6 months
**F2**	**A2**	Both **P2 and P3**	No	Consultation	In person	< 6 months
	**A3**	Both **P2 and P3**	No	Consultation	In person	< 6 months
**F3**	**A4**	Mother **P4**	Yes	Consultation	In person	< 6 months
	**A5**	Mother **P4**	No	Consultation	In person	< 6 months
**F4**	**A6**	Mother **P5**	Yes	Consultation	By videoconference	> 6 months
**F5**	**A7**	Mother **P6**	Yes	Tele-consultation	By videoconference	> 6 months
**F6**	**A8**	Mother **P7**	No	Consultation	By videoconference + phone	> 6 months
**F7**	**A9**	Mother **P8**	Yes	Tele-consultation	By phone	> 6 months
**F8**	**A10**	Mother **P9**	Yes	Consultation	In person	< 6 months

A = Adolescent

**Fig 1:**
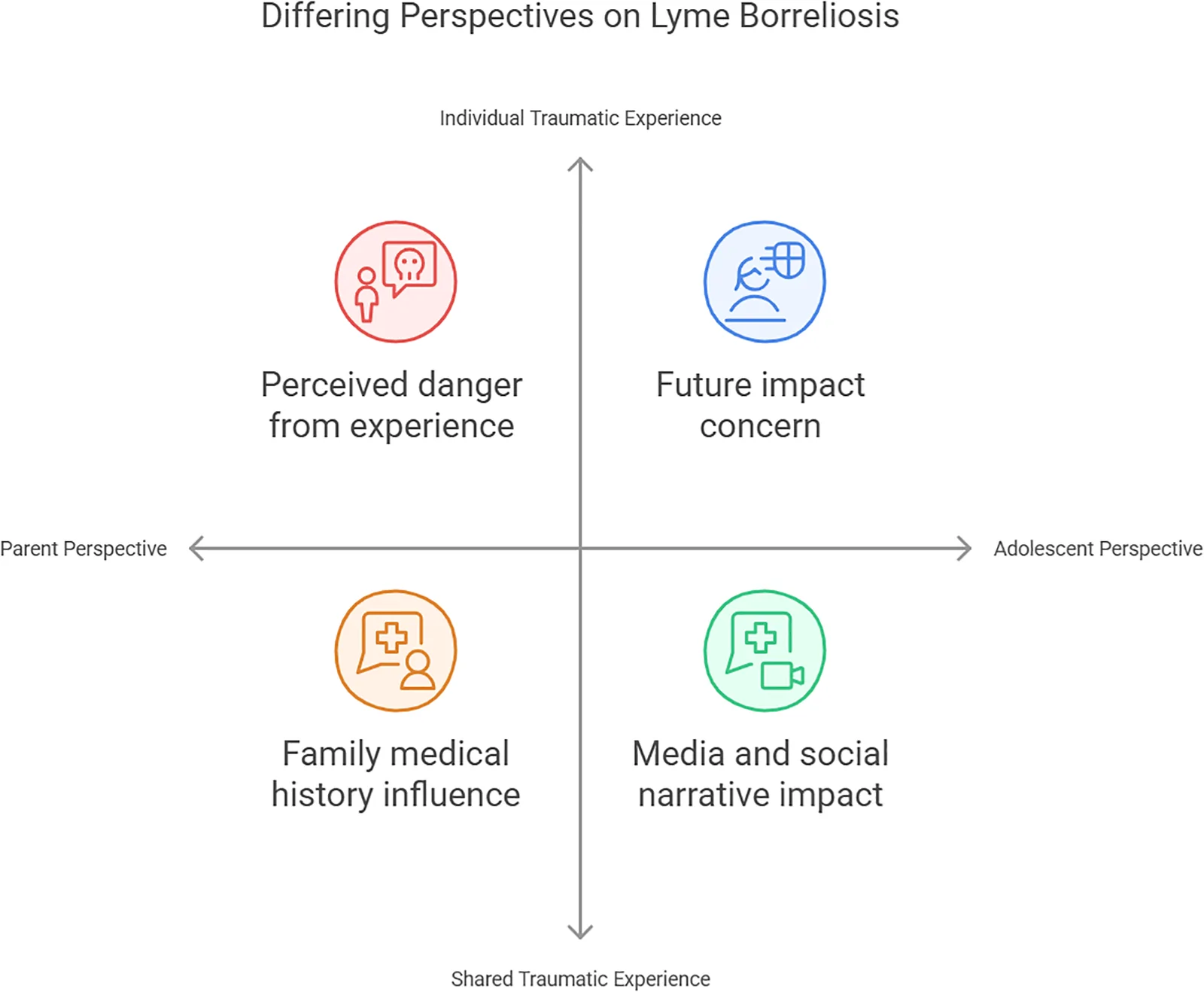
Mind map of theme 1: A personal experience of LB influenced by family history.

**Fig 2:**
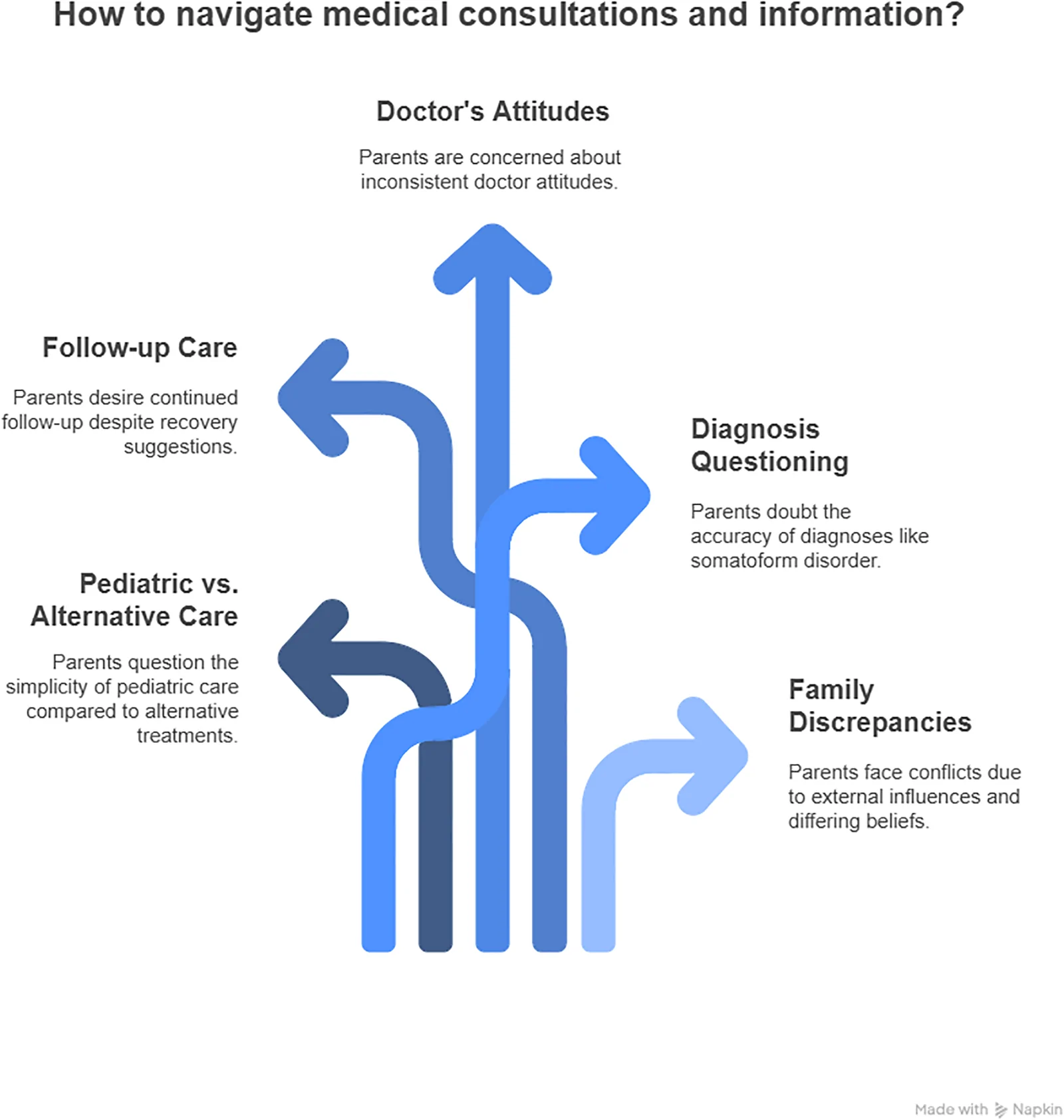
Mind map of theme 2: An ambivalent experience of medical discourse.

**Fig 3:**
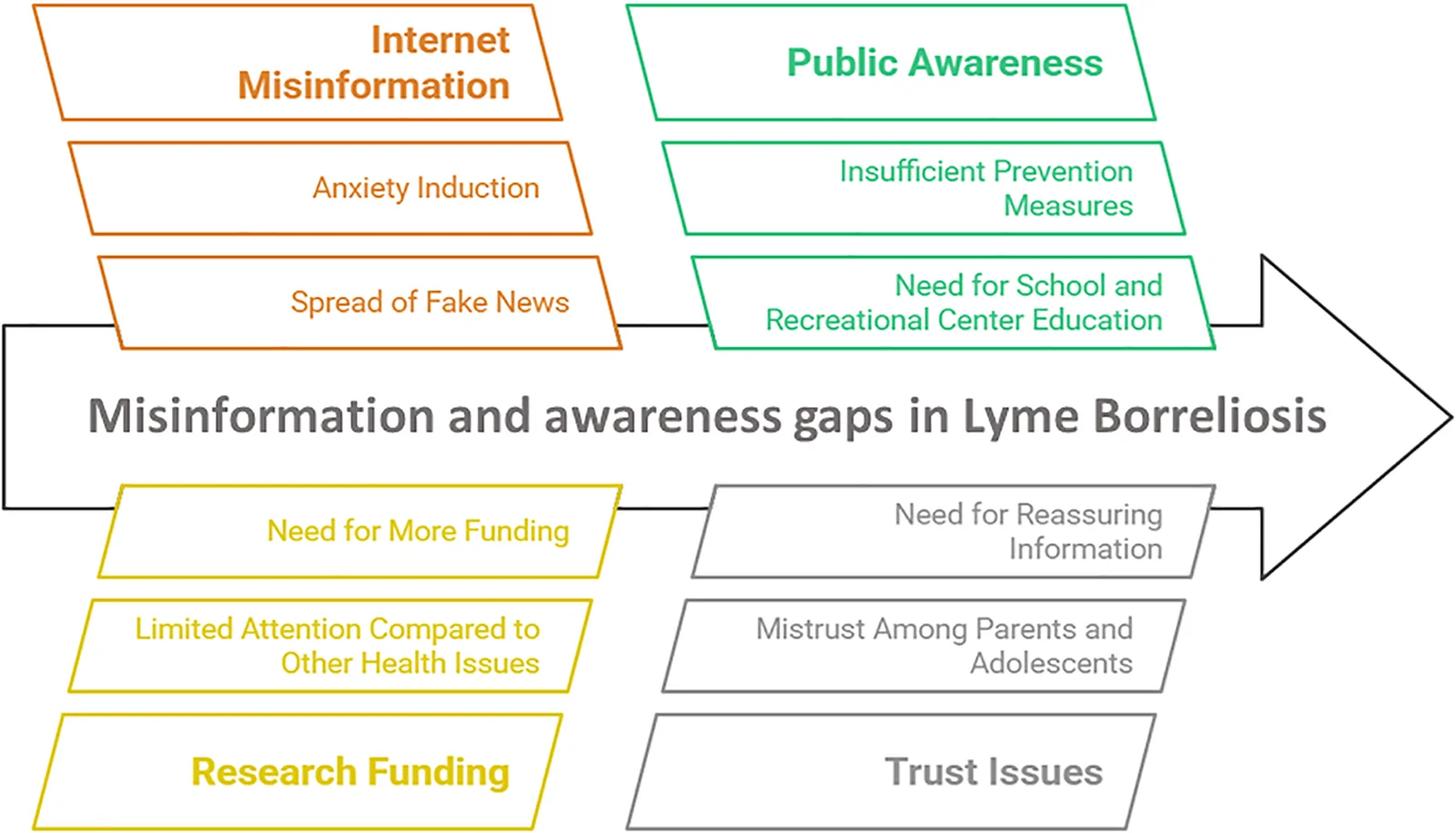
Mind map of theme 3: The need to improve knowledge about LB to prevent misinformation.

The word cloud provided a visual representation of word occurrences answering the question “*Can you give me three words that come to mind when you think about LB?*” to gain exploratory insight into the data (S1 Fig), and row data are presented in S2 Table. The most frequent word was “tick” and the most frequent symptom was “pain”, reflecting the importance of the symptoms in the perception of LB. We identified 1340 adverbs among “few”, “good”, “often”, “sometimes”, “a lot”, “too much/too many” etc (S3 Table). Open coding identified 809 codes. Synonyms and terms from the same lexical field were grouped together in a first step (211 final codes), before proceeding with the thematic analysis by non-predefined themes and sub-themes (Table 2 and Figs 1–3).

**Table 2:** Themes, sub-themes, and codes.

Themes	Sub-themes	Number of final codes*
Theme 1: A personal experience of LB influenced by family history	1.1 Intra-familial transmission of negative representations of Lyme borreliosis	20
	1.2 Confrontation of contradictory family experiences	47
	1.3 Tensions and family dynamics around diagnosis and management of Lyme borreliosis experience	26
	1.4 Knowledge and experiences of tick bites	20
	1.5 Negative social representations of ticks	13
	1.6 High-risk locations, behaviors, and environments	5
	1.7 Prevention: some knowledge and a sense of ineffectiveness	18
Theme 2: An ambivalent experience of medical discourse	2.1 Trust in medical competence and diagnosis	18
	2.2 Seeking a second specialised opinion	6
	2.3 Expectations for better care	20
Theme 3: The need to improve knowledge about LB to prevent misinformation	3.1 The need for better public information	17
	3.2 Issues related to misinformation (fake news)	1

*Open coding identified 809 codes. Synonyms and terms from the same lexical field were grouped together in a first step ending in 211 final codes

The pre-interviews provided additional insights for each theme and helped refine the subthemes. The pre-interviews contributed 23 additional grouped codes. As no fathers were included in the main interviews, it seemed valuable to retain the pre-interviews conducted with two fathers, particularly because they raised questions about the diagnosis of LB and related care pathways. It also enabled to get perspectives from a whole family (father, mother, and 2 children). These new codes helped shed light on complex family dynamics that had until then been barely visible in our analysis. It revealed the existence of intra-familial conflicts and power dynamics among family members around the management of LB. These dynamics manifested notably through tensions related to the legitimacy of the diagnosis, an imbalance in the distribution of parental roles, or the repositioning of the paternal role in the face of a mother taking control of the child’s health. For example, a case of self-medication imposed by a parent highlighted how social representations and parental perceptions of the disease could generate a distressing experience for the entire family, including the teenager, who was led to feel guilty if they questioned their own care. Furthermore, these interviews enriched our understanding of the participants’ emotional experiences, with direct expressions of distress and loneliness, as well as feelings regarding healthcare management in France and the gap between alternative and official therapeutic approaches. Finally, the recognition of reference centers emerged as a new and structuring element, absent from the earlier interviews.

The interview guide did not change substantially after the pre-interviews, so the same guide was retained for the interviews. However, these preliminary discussions helped reinforce its validity.

### 3.1. Theme 1: A personal experience of LB influenced by family history

Parents often perceived LB as an “*incurable*” (P5), “*sneaky*” (P3), and “*dangerous*” (P6) condition, based on their own experiences either as patients or caregivers: “*My husband and I had Lyme disease too*” (P5). Some parents had an ambivalent perception of LB: “*[LB] is at the same time, it’s not a serious illness, like in my case thanks to good care, honestly, I didn’t feel anything, I took two weeks of antibiotics and that was it. But it can also be really debilitating I think, with some really severe forms. Paralysis, stories of people in wheelchairs…”* (P4). LB was also seen as a “*subject of debate*” (P1) as it is “*not so well known with a lot of crazy theories*” (P1). In contrast, most adolescents had a relatively non-traumatic experience with the illness: “*I was never really worried. Sometimes I even forgot I had it, so there you go. I really didn’t care about it at all, actually.”* (A10). Table 3 illustrates some opposite codes between adolescents and their parents. Although the experiences of parents and adolescents were different, key elements emerged:

**Table 3:** Opposite codes between adolescents and parents.

Theme	Sub-theme	Adolescents	N adolescents	Parents	N parents
Theme 1: A personal experience of LB influenced by family history	1.1 Intra-familial transmission of negative representations of Lyme borreliosis	Lyme disease perceived as not very concerning, non-traumatic experience (“doesn’t really bother me” A7, “not a traumatic experience” A10, “it’s nothing serious” A7, A8)	7 (A2, A3,A4,A5 A6, A7, A8)	Lyme disease perceived as serious, dangerous, incurable (“incurable disease” P5, “dangerous” P5, “you could die” P5, “degenerative disease” P6, “can turn into a nightmare” P3)	4 (P3, P5, P6, P7)
	1.2 Confrontation of contradictory family experiences	Little or no emotional impact (“not a distressing experience” A7, “not worried” A10, “I’m fine now” A7, A8)	6 (A2, A3, A6, A7, A8, A10)	Strong emotional impact (“kind of depression” P2, “a lot of stress” P5, P7, P8, “complete uncertainty” P7, “helpless” P7)	6 (P2, P3, P5, P6, P7, P8)
	1.3 Tensions and family dynamics around diagnosis and management	Feeling of guilt, passive management (“it’s annoying to take antibiotics every day…”, A8)	5 (A1, A2, A3, A7, A8)	Power dynamics, role shifts, self-medication (“regrets not having taken control from the start”, P5)	5 (P1, P3, P4, P5, P7)
Theme 2: An ambivalent experience of medical discourse	2.1 Trust in medical competence and diagnosis	Passive trust	7 (A1, A2, A3, A6, A7, A8, A9)	Trust but questioned	7 (P1, P2, P4, P5, P6, P7, P9)
	2.2 Seeking a second specialized opinion	Little involvement in decisions	7 (A1, A2, A3, A6, A7, A8, A9)	Active search for specialists	7 (P1, P3, P4, P5, P6, P7, P9)
	2.3 Expectations for better care	Need for clearer explanations	5 (A1, A3, A8, A9, A10)	Dissatisfaction with care delays/absence of follow-up	7 (P1, P3, P4, P5, P6, P7, P8)
Theme 3: Need to improve knowledge about LB to prevent misinformation	3.1 Need for better public information	Desire for reassuring information	4 (A1, A6, A8, A10)	Need for awareness campaigns	7 (P1, P2, P4, P5, P6, P7, P9)
	3.2 Misinformation (fake news)	Peer rumors and confusion	3 (A1, A6, A10)	High exposure to fake news	7 (P1, P2, P4, P5, P6, P7, P9)

- **A shared perception of LB as mysterious and dormant,** with the potential to suddenly impact them in the future: “*Maybe in the future, LB could have a significant impact. Because right now, I might have gotten used to it and don’t perceive it*” (A7). Some adolescents emphasized the role of narratives, whether accurate or based on rumors, shared by the media or their social circles: “*I heard some rumors... it could completely paralyze me*” (A6).
- **The weight of family inheritance and medical history** sometimes led to feelings of abandonment by healthcare professionals or a lack of understanding: *“ It’s all a bit unclear, really! And you get the feeling that… well, that you’re not necessarily taken seriously. It’s psychosomatic, fine! But that doesn’t cover everything!”* (P7). For adolescents, it leads to concerns that their complaints might be doubted by their parents. Nonetheless, all the parents expressed unwavering support for their child.

### 3.2. Theme 2: An ambivalent experience of medical discourse

All the families seemed satisfied with their medical consultations at the two hospitals involved in our study and with the information they received. However, this information was often questioned for several reasons:

- **Discrepancy between pediatric and alternative care approaches** was noticeable between the simplicity of the care proposed at specialized centers compared to the one offered in alternative care paths: “*We even did, uh, a vet test! We even had a third test, another one! that we sent to Germany*,” “*He had given him a list of medications, quite impressive! He had to take at least ten to fifteen every day*” (P8). An adolescent mentioned a lack of understanding regarding her alternative treatment and the absence of improvement of her condition: “*Since I was still tired after two years, it was [my father] who told me to stop with the Lyme doctor, and he referred me to the reference center.*” (A1) Four families had chosen these alternative care pathways: F1, F2 and F7 consulted “Lyme doctors” with non-recommended treatments and diagnostic tests performed in Germany or the USA; F8 used self-medication with vitamins.
- **Discontinuation of follow-up in the absence of symptoms** was questioned by parents (P4, P5, P6) and they suggested to continue follow-up even if recovery was mentioned: “*I’ve never heard that it heals spontaneously!*” (P5).
- **Destabilizing attitudes of previous doctors** was responsible for doubts: “*We’ve just consulted two [general practitioners]. One who took the problem somewhat lightly, and the other who got agitated, so what now?*” (P9). Two families (F6 and F8) were destabilized by opposite discourses and a feeling of not being taken seriously.
- **Questioning of the medical diagnosis** was frequent, particularly the diagnosis of somatoform disorder: *“It is a bit unclear, really! And you get the feeling that… that you’re not taken seriously. I strongly believe in psychosomatics! Fine! But it doesn’t explain everything, all the time!*” (P7). The term *psychosomatic* was sometimes interpreted as a label given by doctors to someone who was believed to be fabricating their symptoms. Four families found that diagnosis was unclear (F1, F2, F6, F8).
- **Discrepancies within the family** were shaped by external influence: **“***It was a friend of my wife who ‘made’ the diagnosis of Lyme disease, if you will*” (P1). Some parents noted that their own parents believed the child was healthy and blamed them for “*imagining illness*” (P2). Tensions also arose within families over how to care for the child. One parent, dissatisfied with French medical care, turned to an American “Lyme doctor”. Another parent initiated alternative treatment at home, leading to family pressure and emotional strain. Some parents regretted not taking a more active role in their child’s healthcare earlier: *“I regret not having taken things into my own hands from the start. It’s hard to feel sidelined and powerless in the care of your own child”* (P1). Some adolescents expressed guilt over the situation, feeling burdened by the emotional reactions of their parents: *“Honestly, it sucks taking antibiotics every day, seeing your mother cry, hearing that you’re going to die and all that”* (A3).

### 3.3. Theme 3: The need to improve knowledge about LB to prevent misinformation

The majority of respondents expressed concerns about using the internet due to the risk of encountering incorrect information: “*So I refuse to look things up on the internet! Because there’s everything and anything!*” (P7). Some participants highlighted the spread of “*fake news*,” which contributes to increased anxiety: “*When you see things on the internet, most of the time they’re false! On top of that, there is not necessarily any reliable information, so it makes people more anxious...*” (A10). This sense of mistrust toward online information was shared by both parents and adolescents. Others wished that existing reference centers were more prominently promoted, especially online.

Others expressed a desire for increased funding for research on LB, noting that it receives less attention compared to other health crises like COVID-19: “*To increase research, we need funding! But to get funding, it needs to be an interesting disease!*” (P8).

Both parents and adolescents also emphasized the need to improve public awareness about LB and its prevention, particularly in forested areas frequented by children and school groups: “*if we were given more information about [LB] and it was better explained, it could have been reassuring*” (A10). Lastly, even though some were familiar with tick prevention due to their outdoor activities, many expressed a lack of knowledge about the risks associated with ticks: *“I’ve had lots of ticks on me. I removed plenty of them! And I’ve already seen a bull’s-eye rash (laughs). I didn’t know much more than that!”* (A1).

## 4. Discussion

### 4.1. Grounded theory explanatory model

**Fig 4:**
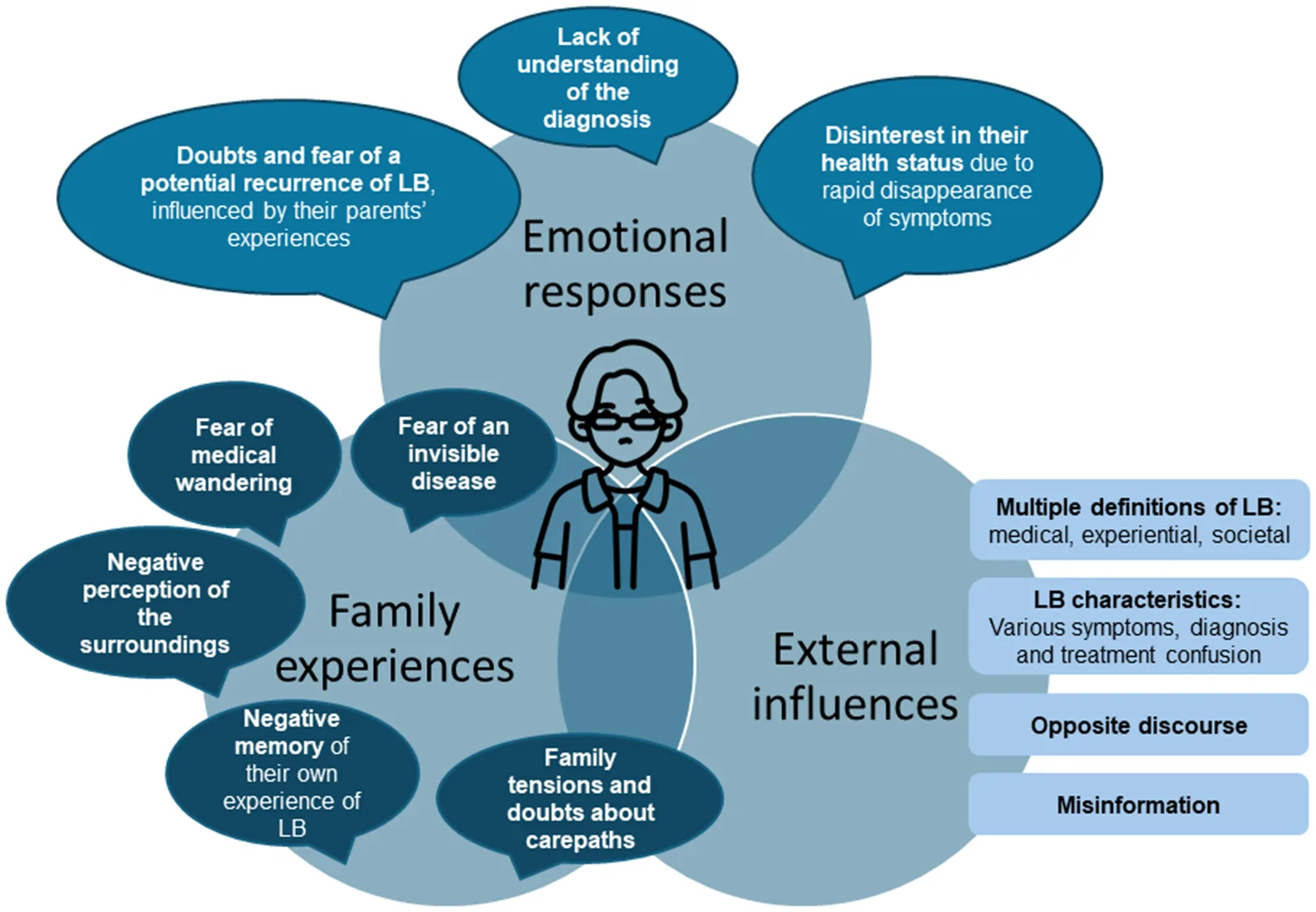
Explanatory model of perceptions, social representations, and experiences of adolescents presenting with suspected Lyme borreliosis (LB) and their parents.

Based on the results presented, an explanatory model has been proposed to theorize the relationships between perceptions, representations, and lived experiences in the context of suspected LB in adolescents and their families (Fig 4). Adolescents’ experiences and representations were shaped by their emotions, past family experiences, and external influences, such as misinformation and difficulties in verifying sources, the difficult-to-understand diagnosis of LB, and the conflicting discourses.

### 4.2. The invisible disease

A contrasting result appeared in our study: most of the adolescents answered “pain” when asked to provide three words that come to mind regarding LB, yet none had actually experienced pain during their acute LB phase. This finding aligns with data from published pediatric cohorts, where pain and prolonged disabling symptoms are rarely reported [7–9,35,36]. It underscores the influence of external knowledge. Indeed, LB was primarily perceived by our patients as a dormant disease, characterized by an invisible risk, as has been previously demonstrated [10,14,15]. One key argument from patient advocacy groups is that *Borrelia burgdorferi* is an “*invisible*” bacterium, imperceptible to the naked eye and even challenging to detect under a microscope. Additionally, serological diagnoses vary depending on the disease’s stage. Therefore, LB is often regarded as an invisible disease due to its difficulty in diagnosis. This silent side, free from symptoms, is associated with the belief that the disease might awaken one day and result in acute and chronic pain, which could be responsible for anxiety—especially as teenagers might fear pain. A systematic review of the literature highlighted that chronic pain engages brain regions critical for cognitive and emotional assessment, implying that this component of pain may be associated with a past medical history of pain, its perception, its representation, and personal experience [37]. Moreover, Albinni et al. demonstrated that acute pain during childhood shapes pain perception and influences its experience in adulthood, sometimes leading to fear and anticipation [38]. The pain experience of parents may also have psychological, behavioral, and developmental consequences on their child’s perception of pain.

### 4.3. The social representations of LB in the care trajectory

Anglo-Saxon terminology enables to approach illness from various angles: the social perspective, termed “sickness”; the biological perspective, called “disease”; and the individual experiential perspective, known as “illness” [39]. The social representations of LB can sometimes differ between patients and the medical community [10,15,17], because some patients feel that doctors fail to understand their symptoms and refuse treatment, even though their daily lives are significantly impacted [10,14,40]. In response to these persistent symptoms and their lack of management, advocacy groups were started to push for the recognition of “chronic” Lyme disease [41,42], which have become very active on the internet and social media [43,44]. Disease representations are social and cultural constructs that all societies develop to explain the meaning, origin, and causes of illnesses, particularly those that confront death and helplessness [45]. Therefore, it is essential to consider the various social representations of LB that arise from personal and familial experiences, irrespective of beliefs regarding its chronicity [42].

From a psychological standpoint, the unpredictability of a disease has been linked to an increased risk of psychological difficulties in adolescents, e.g., with a parent suffering from cancer [45]. Moreover, adolescents coping with chronic or uncertain illness use problem-focused, emotion-focused, and avoidance strategies [46–48]. Active coping, including problem-solving, emotional expression, and seeking social support, is linked to better adjustment and quality of life, while avoidance often increases stress and anxiety over time. Uncertainty about the illness strongly influences coping choices. Effective coping typically combines acceptance, proactive problem-solving, emotional regulation, and support from family, peers, or healthcare professionals. These findings underscore the importance of interventions that strengthen adaptive coping skills and help adolescents manage the challenges and unpredictability of chronic conditions.

The impact of LB unpredictability was also highlighted in our analysis, conducted through the lens of the sociology of experience [49]. Our study revealed a variety of disease trajectories for LB. The concept of a disease trajectory “associates the evolution of the pathology with all the actions taken over time by various actors involved in managing its course, treating it, and shaping it” [49]. Consequently, the trajectory of care can significantly influence the ways in which LB is perceived.

### 4.4. Care pathways: from negotiation to satisfaction?

This study demonstrated that, within these challenging consultations, there was a process of negotiation between patients, parents, and healthcare practitioners. The interviews revealed an absence of tension in some families, while others experienced conflict due to differing perceptions and experiences of LB. Family tensions related to the management of LB stem primarily from parents’ frustration with the medical system, disagreements over which treatments to pursue, and the influence of people outside the family. These factors generate internal conflicts and affect the psychological well-being of the children, which aligns with findings from other studies [42,43]. Both parents and children expressed a shared desire for improved communication and better dissemination of information. Co-construction of knowledge about LB took place between parents and doctors and also between adolescents and healthcare providers. Respondents particularly appreciated the patient-centered approach adopted by healthcare practitioners, along with their reassuring demeanor and attentive listening. This point of satisfaction was already described in previous studious in adults with a suspicion of LB [14,15].

### 4.5. Implications of the study for national public health policy: struggle against misinformation

To improve relationships between patients, adolescents, and healthcare practitioners, it would be beneficial to develop specialized training for doctors on LB and, more broadly, on TBD and post-infectious syndromes (persistent symptoms following infections like LB or COVID-19, or others) [17,50]. This training should also focus on the management of subjective, disabling symptoms, such as pain, to prevent confusion among patients that arises from inconsistent messaging by healthcare professionals. Harmonizing key messages about LB is essential.

Additionally, increasing the spread of information about LB and tick bite prevention among adolescents is necessary to reduce the risk of tick bites and, consequently, the risk of infection [50].

Finally, efforts to share verified information through media, social networks, and the internet are crucial, as these platforms influence norms, thought systems, and values regarding diseases [43,44,51–55]. They also provide visibility for so-called “chronic” diseases. Non-expert resources often “fill the gap” left by healthcare practitioners, sometimes giving individuals the impression that they can address their medical needs independently. These informational materials are frequently perceived as revealing truths and reflecting the population’s anxieties and fears [51]. The internet could also play a role in widely disseminating verified and reliable information. However, the overabundance of information or misinformation can lead to the spread of “fake news” and its associated dangers, particularly highlighted by respondents in our study. It is critical that information shared online be verified, evidence-based, and properly referenced.

### 4.6. Strengths and limitations of the study

To our knowledge, this is the first study to focus on the perceptions, social representations, and experiences of adolescents consulting for suspected LB. The dyadic approach used in this study was crucial for gaining a deeper understanding of interactions by examining the perspectives of both members of the dyad. It allowed us to identify relational dynamics—how each individual influences and is influenced by the other. This comprehensive and nuanced view of shared experiences helps reveal the complexities of social interactions and individual experiences. A dyadic qualitative approach was adopted, as the phenomenon under study was inherently relational and could not be fully understood through individual perspectives alone. This design enabled the exploration of how experiences, meanings, and decisions were co-constructed through interaction between participants. By conducting joint interviews, we were able to capture relational dynamics *in situ*, including agreements, disagreements, and negotiation processes, as well as asymmetries in knowledge and positioning. For example, we identified new sub-themes such as sub-theme 1.3, which highlights tensions and family dynamics around diagnosis and management of LB experience.

The dyadic approach also facilitated the identification of convergent and divergent perspectives within the same relational context, providing a more nuanced understanding of participants’ experiences. This was particularly relevant in the context of LB, where diagnostic processes and care pathways are often shaped by interactions between patients, their relatives, and healthcare professionals.

Since, only two fathers participated in the study, it is important to include more input from fathers that could provide a better understanding of the family structure and its role in shaping social representations of LB. This asymmetry might introduce a bias into conclusions about the family’s role in shaping adolescents’ representations of LB.

Furthermore, pilot interviews were included, with some potential bias that we tried to mitigate. The exploratory pre-test interviews did not differ in structure from the subsequent interviews and received equal attention during the analysis. While early interviews may have produced slightly less detailed data due to the interviewer’s developing experience, this limitation was balanced by the inclusion of fathers at this stage, which contributed to a more representative sample. Data saturation was reached at the 18^th^ participant, highlighting the complexity of the social representations studied using a dyadic approach. The third theme emerged during the first interview. The targeted questions in the guide contributed to the early identification of the themes at the start of the inclusion period. However, they were further explored from different perspectives thanks to these 19 interviews. No new codes appeared after the 18th interview. In line with grounded theory methodology, data collection and analysis were conducted iteratively, with constant comparison guiding the development of categories. While theoretical saturation was sought, it was considered in pragmatic terms. Data collection was stopped when categories were sufficiently developed in relation to the study aims and when additional data no longer contributed substantially new insights. This approach is consistent with the notion of “theoretical sufficiency,” acknowledging that full saturation may be difficult to achieve in practice. We assume that maybe we could have found more codes, especially as this issue is poorly studied (very few studies with adolescents and LB).

The affiliation of the authors with a TBD reference center may have influenced data collection and interpretation that we tried to mitigate. SC conducted the interviews and coded the transcripts. She had no prior contact with the patients in routine care, as she is a researcher in medical anthropology. All interviews were anonymized by SC before being independently coded by GP. Nevertheless, as some of the authors are physicians with expertise in the management LB, the interpretation of the data may have been influenced by their clinical experience. Therefore, a latent analytical approach was adopted to better understand participants’ perspectives in a comprehensive manner and to highlight practical implications for healthcare relationships. We also explicitly identified our own interpretive positions to support a critical analysis of the data, including reflexivity regarding our subjectivity. The perspectives were compared to identify convergences and divergences, leading to a final consensus.

## 5. Conclusion

The results revealed that both personal and collective experiences significantly influence the perceptions of adolescents and their parents regarding LB. Disinterest in LB was the most commonly shared sentiment among adolescents, even if other feelings were also identified (fear, anxiety). For parents, their roles ranged from negotiating with healthcare practitioners to acting as a caregiver for their child, sometimes leading to internal tensions. There was also a call for clear, accessible, and easy-to-understand information about the management of LB toward patients and healthcare providers, especially general practitioners. Future studies could benefit from considering all perspectives on LB, analyzing them through an interactionist approach that incorporates the perceptions of doctors, patients, and their broader social contexts.

## Supporting information

S1 Table Interview guide with discussion issues. (DOCX)

S2 Table Table precising the word cloud with frequency of the top terms answering the question “Can you give me three words that come to mind when you think about LB?”. (DOCX)

S3 Table Adverbs frequency among the interviews. (DOCX)

S1 Fig Word cloud answering the question “Can you give me three words that come to mind when you think about LB?”. (TIFF)

## Abbreviations

A: Adolescent
COVID-19: CoronaVIrus Disease-19
LB: Lyme borreliosis
P: Parent
TBD: Tick-Borne Diseases
