# Supplementary material for: Perceptions, social representations, and experiences of suspected Lyme borreliosis among adolescents and their parents in France: A multicenter study
Source: PLoS One. 2026 Sep 21;21(9):e0357185. doi: 10.1371/journal.pone.0357185 (PMC13592628; doi:10.1371/journal.pone.0357185)
Supplement: Supplementary file 1 [file pone.0357185.s001.docx]

***S1 Table.*** *Interview guide with discussion issues*

| **Themes** | **Adolescent** | **Parent** |
| --- | --- | --- |
| **Care pathway** |  Can you explain your medical journey since the onset of the first symptoms? (e.g. doctors consulted, hospital care, general practitioners, alternative medicine?)   How long has LB been a part of your discussions?   Have you used other, more alternative treatments? Did these methods help you?  or Have you consulted a "Lyme doctor"? What was that experience like (doctor-patient relationship, prescriptions, explanations, etc.)? Have you seen any other doctors? | |
| **Knowledge of LB** |  How would you define LB in your own words?   Did you know about LB before suspecting that you might have it? If so, how?   Have you researched this disease on your own? If so, what tools or resources did you use? (e.g., Internet, literature) | |
| **Social Representations of LB** |  How do you perceive LB? What are your thoughts on it?   What three words come to mind when you think about LB? | |
| **Disease Management** |  How are you managing LB in the context of the COVID-19 health crisis over the past two years?   How do you handle the disease on a daily basis? Do you have any daily care routines? If so, what do they involve?   What was the diagnosis process like? |  How did you manage your child's health during the COVID-19 crisis? Were you able to get the necessary support? |
| **Discussion of LB within the family** |  Do you talk about LB with those around you? Do they support you? |  Do you discuss LB with your child? How?   Who was the first to consider LB as a possibility?   How are you and your child involved in managing LB? |
| **General feelings** |  What are your overall feelings about the care you have received?   What are your thoughts on the diagnosis process? | |
| **Questions for parents regarding LB** |  |  Have you had a similar experience with LB as your child?   Have you had or suspected LB yourself?   What is your medical history?   How do you feel about your experience with LB today? |
| **Feelings during the interviews** |  How do you feel today about your current health situation?   What are the main takeaways from your experience? What impact has it had on your life? | |
| **Collective questions about LB** |  How has this illness affected your daily life together?   What are your overall feelings about the caregiving experience?   Have you received support and understanding from medical staff, family and friends, educational staff, or colleagues?   Have patient associations provided you with any help? | |
