# Supplementary material for: Perceptions, social representations, and experiences of suspected Lyme borreliosis among adolescents and their parents in France: A multicenter study
Source: PLoS One. 2026 Sep 21;21(9):e0357185. doi: 10.1371/journal.pone.0357185 (PMC13592628; doi:10.1371/journal.pone.0357185)
Supplement: Supplementary file 2 [file pone.0357185.s002.docx]

***S2 Table.*** *Table precising the word cloud with frequency of the top terms answering the question “Can you give me three words that come to mind when you think about LB?"*

| Words | Total N | Adolescents N | Parents N |
| --- | --- | --- | --- |
| Tick | 9 | 6 | 3 |
| Pain | 7 | 4 | 3 |
| Fatigue | 5 | 2 | 3 |
| Nothing | 4 | 4 | 0 |
| Wandering | 3 | 1 | 2 |
| Mysterious | 2 | 0 | 2 |
| Benign | 2 | 0 | 2 |
| Insidious | 2 | 1 | 1 |
| Paralysis | 2 | 1 | 1 |
| Disabling | 2 | 0 | 2 |
| Long | 1 | 1 | 0 |
| Incomprehensible | 1 | 1 | 0 |
| Far-fetched | 1 | 0 | 1 |
| Invisible | 1 | 1 | 0 |
| Asymptomatic | 1 | 1 | 0 |
| Rare | 1 | 0 | 1 |
| Serious | 1 | 0 | 1 |
| Destructive | 1 | 0 | 1 |
| Medication | 1 | 1 | 0 |
| Erythema | 1 | 1 | 0 |
| Variable | 1 | 0 | 1 |
| Infection | 1 | 1 | 0 |
| Dangerous | 1 | 0 | 1 |
| Everywhere | 1 | 0 | 1 |
| Hard to treat | 1 | 0 | 1 |
| Hard to detect | 1 | 0 | 1 |
| Headache | 1 | 1 | 0 |
| Research | 1 | 0 | 1 |
