# Supplementary material for: Perceptions, social representations, and experiences of suspected Lyme borreliosis among adolescents and their parents in France: A multicenter study
Source: PLoS One. 2026 Sep 21;21(9):e0357185. doi: 10.1371/journal.pone.0357185 (PMC13592628; doi:10.1371/journal.pone.0357185)
Supplement: Supplementary file 3 [file pone.0357185.s003.docx]

***S3 Table.*** *Adverbs frequency among the interviews*

| Adverbs | Frequency |
| --- | --- |
| Few | 537 |
| Good | 230 |
| A lot | 142 |
| Too much/Too many | 125 |
| Never | 74 |
| Less | 54 |
| Many | 50 |
| Better | 47 |
| Often | 40 |
| Sometimes | 21 |
| Very good | 20 |
