# Supplementary material for: Perceptions, social representations, and experiences of suspected Lyme borreliosis among adolescents and their parents in France: A multicenter study
Source: PLoS One. 2026 Sep 21;21(9):e0357185. doi: 10.1371/journal.pone.0357185 (PMC13592628; doi:10.1371/journal.pone.0357185)
Supplement: Peer review history

# Peer review: Decision Letter 0

**DOI:** 10.1371/journal.pone.0357185.r001

**Authors:** Stephen M. Rich

31 Mar 2026

Dear Dr. Raffetin,

Provide intermediate analytic scaffolding showing how 809 codes were consolidated into three themes. This may include a codebook excerpt or table of subthemes, and add frequency language ("most," "several," "a minority") with approximate counts to characterize pattern distribution.

Both reviewers questioned the decision regarding pre-test family inclusion. Clearly state in the Results whether these interviews contributed substantively to final themes, whether guide changes occurred afterward, and whether sensitivity analyses were conducted.

The reliance on a single reference (Laplantine) for "social representations" is insufficient given its centrality to your study. Engage with foundational social representations literature and more recent illness representation scholarship.

Expand discussion of how your affiliation with the TBD Reference Centre may have influenced data collection and interpretation, per COREQ requirements.

Ensure all participant quotations are complete, clearly translated, and properly attributed. Truncated excerpts compromise assessment of interpretive rigor.

Specify participant contribution, whether responses came from adolescents, parents, or both, and consider presenting top terms in tabular format rather than relying solely on the word cloud.

The manuscript requires thorough editing by a native English speaker to address errors affecting readability. Also, please consider including more recent literature on health infodemics (2021–2024) and adolescent coping with chronic/uncertain illness and correct incomplete references per PLOS ONE style.

Please submit your revised manuscript by May 15 2026 11:59PM. If you will need more time than this to complete your revisions, please reply to this message or contact the journal office at plosone@plos.org. When you're ready to submit your revision, log on to https://www.editorialmanager.com/pone/ and select the 'Submissions Needing Revision' folder to locate your manuscript file.

Please include the following items when submitting your revised manuscript:

- A letter that responds to each point raised by the academic editor and reviewer(s). You should upload this letter as a separate file labeled 'Response to Reviewers'.
- A marked-up copy of your manuscript that highlights changes made to the original version. You should upload this as a separate file labeled 'Revised Manuscript with Track Changes'.
- An unmarked version of your revised paper without tracked changes. You should upload this as a separate file labeled 'Manuscript'.

If you would like to make changes to your financial disclosure, please include your updated statement in your cover letter. Guidelines for resubmitting your figure files are available below the reviewer comments at the end of this letter.

If applicable, we recommend that you deposit your laboratory protocols in protocols.io to enhance the reproducibility of your results. Protocols.io assigns your protocol its own identifier (DOI) so that it can be cited independently in the future. For instructions see: https://journals.plos.org/plosone/s/submission-guidelines#loc-laboratory-protocols. Additionally, PLOS ONE offers an option for publishing peer-reviewed Lab Protocol articles, which describe protocols hosted on protocols.io. Read more information on sharing protocols at https://plos.org/protocols?utm_medium=editorial-email&utm_source=authorletters&utm_campaign=protocols.

As the corresponding author, your ORCID iD is verified in the submission system and will appear in the published article. PLOS supports the use of ORCID, and we encourage all coauthors to register for an ORCID iD and use it as well. Please encourage your coauthors to verify their ORCID iD within the submission system before final acceptance, as unverified ORCID iDs will not appear in the published article. *Only*  the individual author can complete the verification step; PLOS staff *cannot*  verify ORCID iDs on behalf of authors.

We look forward to receiving your revised manuscript.

Kind regards,

Stephen M. Rich, MS, PhD

Academic Editor

PLOS One

Journal Requirements:

When submitting your revision, we need you to address these additional requirements.

1. Please ensure that your manuscript meets PLOS ONE's style requirements, including those for file naming. The PLOS ONE style templates can be found at

https://journals.plos.org/plosone/s/file?id=wjVg/PLOSOne_formatting_sample_main_body.pdf and

https://journals.plos.org/plosone/s/file?id=ba62/PLOSOne_formatting_sample_title_authors_affiliations.pdf

2. In this instance it seems there may be acceptable restrictions in place that prevent the public sharing of your minimal data. However, in line with our goal of ensuring long-term data availability to all interested researchers, PLOS’ Data Policy states that authors cannot be the sole named individuals responsible for ensuring data access (http://journals.plos.org/plosone/s/data-availability#loc-acceptable-data-sharing-methods).

Data requests to a non-author institutional point of contact, such as a data access or ethics committee, helps guarantee long term stability and availability of data. Providing interested researchers with a durable point of contact ensures data will be accessible even if an author changes email addresses, institutions, or becomes unavailable to answer requests.

Before we proceed with your manuscript, please also provide non-author contact information (phone/email/hyperlink) for a data access committee, ethics committee, or other institutional body to which data requests may be sent. If no institutional body is available to respond to requests for your minimal data, please consider if there any institutional representatives who did not collaborate in the study, and are not listed as authors on the manuscript, who would be able to hold the data and respond to external requests for data access? If so, please provide their contact information (i.e., email address). Please also provide details on how you will ensure persistent or long-term data storage and availability.

3. Your ethics statement should only appear in the Methods section of your manuscript. If your ethics statement is written in any section besides the Methods, please delete it from any other section.

4. Please include captions for your Supporting Information files at the end of your manuscript, and update any in-text citations to match accordingly. Please see our Supporting Information guidelines for more information: http://journals.plos.org/plosone/s/supporting-information.

5. If the reviewer comments include a recommendation to cite specific previously published works, please review and evaluate these publications to determine whether they are relevant and should be cited. There is no requirement to cite these works unless the editor has indicated otherwise.

[Note: HTML markup is below. Please do not edit.]

Reviewers' comments:

Reviewer's Responses to Questions

**Comments to the Author**

1. Is the manuscript technically sound, and do the data support the conclusions?

Reviewer #1: Yes

Reviewer #2: Partly

**********

2. Has the statistical analysis been performed appropriately and rigorously?

Reviewer #1: N/A

Reviewer #2: N/A

**********

3. Have the authors made all data underlying the findings in their manuscript fully available?

The PLOS Data policy

Reviewer #1: No

Reviewer #2: Yes

**********

4. Is the manuscript presented in an intelligible fashion and written in standard English?

Reviewer #1: Yes

Reviewer #2: No

**********

Reviewer #1: 1- The Results section reads more like an extended summary than an analytic presentation of grounded-theory outputs. Beyond stating “809 codes” and three themes, the manuscript does not show the intermediate analytic architecture that would allow a reader to judge adequacy and coherence, such as a concise codebook excerpt, illustrative subthemes, or a brief description of how the 809 codes were consolidated into the three themes. Without that scaffolding, the reader cannot assess whether the thematic structure is saturated, internally consistent, or selectively reported.

2- Participant description is thin for a study that explicitly aims to examine perceptions and experiences across a potentially heterogeneous group. Table 1 is helpful as a first pass, but the Results should explicitly quantify key variation variables that were part of the sampling strategy, such as suspected versus confirmed Lyme borreliosis, symptom duration shorter than six months versus longer than six months, and rural versus urban exposure context, rather than leaving these to inference from a small table. This is particularly important given the modest number of adolescents and the risk that a few narratives dominate the synthesis.

3- The inclusion of three pretest families within the analytic dataset needs a more explicit account in the Results, not only in the Methods. If those interviews were retained, it is important to state whether they contributed substantively to the final themes, whether any interview-guide changes occurred after them, and whether a sensitivity check was performed to ensure they did not disproportionately shape early coding trajectories. The current Results do not reassure the reader on this point, especially since saturation is claimed with a small sample.

4- The Results would benefit from sharper within-theme contrasts between adolescents and parents, supported by a consistent strategy for quotation selection. The text asserts divergent experiences, such as parents describing Lyme borreliosis as “incurable” and adolescents expressing relative disinterest, but it remains unclear whether these are dominant patterns, minority voices, or evenly distributed across families. Consider adding brief frequency language that is standard in qualitative reporting, such as “most,” “several,” “a minority,” backed by counts or at least the number of families in which each pattern appeared.

5- The word-cloud result is presented as though it were an empirical “finding,” with “pain” said to be the most frequent word, yet the Results do not specify how many participants contributed to this prompt, whether responses were elicited from adolescents, parents, or both, and whether the prompt was asked once or across the three interviews per family. Given that the Discussion later interprets “pain” as a striking contrast, the Results should be strengthened with clear reporting of the underlying responses, ideally with a short table of the top terms and counts rather than relying on a word cloud as the primary vehicle.

6- Theme 2 is an important contribution, especially the tension between pediatric reference centers and alternative care pathways, and the destabilizing effect of inconsistent medical encounters. However, the Results blend together multiple phenomena, including discontinuation of follow-up, diagnosis contestation, and intrafamilial conflict, without distinguishing which are central versus peripheral. A clearer substructure inside Theme 2, with explicit subthemes and boundaries, would greatly improve interpretability and would reduce the impression that disparate observations have been grouped under an umbrella label.

7- Claims of data saturation and thematic completeness would be more convincing if the Results included concrete indicators: when the third theme first appeared, whether later interviews added only nuance, and how the dyadic third interview influenced or challenged the individual interviews. Since the design involved three interviews per family, the Results should also clarify what unique analytic value came from the dyadic interview, rather than treating the dataset as if it were a single interview per participant.

Reviewer #2: REVIEW REPORT

Manuscript ID: PONE-D-25-58146

Article Type: Research Article

Title: Perceptions, social representations, and experiences of suspected Lyme borreliosis among adolescents and their parents in France: a multicenter study.

Summary: This manuscript presents an original exploratory qualitative study examining how adolescents and their parents perceive, represent, and experience Lyme borreliosis (LB) in the context of a suspected or confirmed diagnosis. It reports data collected through semi-structured interviews analysed using grounded theory with a dyadic approach. The study was conducted in France, in two university hospitals affiliated with a national Tick-Borne Disease (TBD) Reference Centre. Eight families (19 individuals) participated. The title is informative and accurately reflects the study content.

General comments

This study represents a contribution to the literature on LB in paediatrics. The available literature focused on the social representations and lived experiences of adolescents with suspected LB and their parents is scarce. The dyadic methodology is a methodological strength that sets this work apart from single-respondent designs. The grounded theory approach is well-matched to the exploratory research question.

The manuscript is, however, hampered by language issues and several methodological gaps that must be addressed prior to publication.

Strengths

1| Originality and clinical relevance

2| Dyadic methodology

3| Integrative explanatory model

4| COREQ adherence and ethical rigour

Major Concerns

1| Small and potentially biased sample with only 8 families (19 participants), recruited. The sample is likely to overrepresent families with more complex, prolonged, or dissatisfied care trajectories. Theoretical saturation at participant 18 is acceptable for grounded theory but should be contextualized against this recruitment bias.

2| Under-representation of fathers. Only one father participated in the study; all other interviewed parents were mothers. Given that the study places family dynamics and parental tensions at the centre of the analysis, this asymmetry introduces a meaningful bias into conclusions about the family's role in shaping adolescents' representations of LB.

3| Truncated and illegible participant quotations. Multiple participant excerpts in the Results section appear cut off or partially illegible, likely due to formatting issues during file conversion. As direct quotations are the primary form of evidence in qualitative research, this substantially compromises the assessment of interpretive rigour and must be corrected.

4| Insufficient theoretical grounding: The concept of social representations - central to the title and objectives of the study - is grounded almost exclusively in a single reference to Laplantine (ref. 18, a work on the anthropology of illness). The absence of foundational social representations theorists, or more recent literature on illness representations constitutes a theoretical gap that weakens the conceptual framework.

5| Inclusion of pre-test interviews in the final analysis. The 3 pre-test families were included in the final analysis without sufficient methodological justification. Although the authors state that the interview structure did not differ, exploratory interviews conducted while the guide is still under development may yield fewer data. This decision should either be more rigorously justified or its potential impact on the findings explicitly discussed.

6| Insufficient reflexivity statement. COREQ requires a reflexivity statement addressing the researchers' position in relation to the topic studied. The manuscript briefly notes that the investigators belong to Lyme disease reference centre teams - which constitutes a potential perspectival conflict - but does not elaborate on how this positioning may have influenced data collection and analysis.

7| Manuscript requires revision by an English native speaker: the MS contains language errors consistent that can affect readability and, in some instances, interpretability.

Specific comments

Abstract

Line 53 - The term 'carepaths' in line 53 is non-standard; 'care pathways' should be used consistently throughout.

Introduction

Lines 92-97 - The theoretical grounding of social representations relies exclusively on Laplantine (ref. 18). The authors must engage with the foundational social representations literature to justify using this concept as a central theoretical lens. As currently written, the theoretical basis is insufficient for a study that positions social representations as its primary framework.

The study aims are stated clearly, but the rationale for using a dyadic approach specifically (rather than individual interviews with adolescents alone) could be strengthened in this section.

M&M

The reflexivity statement (COREQ items 6-8) is minimal. The investigators are affiliated with a TBD Reference Centre and have previously published qualitative research on LB in adults (ref. 14). This positionality should be explicitly discussed in terms of its potential influence on interview dynamics and data interpretation.

The modality of the joint (family) interview session is not described. Specifically: how was the session structured when adolescents and parents held conflicting views? Was any facilitation protocol applied?

Results

All direct quotes must be presented in full, clearly translated into English, and attributed to the correct participant code.

Discussion

The “invisible disease” is interesting and well-supported by literature. The contrast between adolescents associating LB with 'pain' despite not having experienced it is a noteworthy finding that deserves further elaboration.

“Public health implications” section mixes evidence-based recommendations with speculative statements that lack supporting references. This section should be revised to clearly distinguish findings-based recommendations from general assertions.

Line 313 - contains a typographic error: 'misformation' should be replaced by 'misinformation'.

References

Misinformation and social media (refs. 33, 34, 38, 39): these references date from 2019-2020. Given the manuscript's comparison with COVID-19 and the post-pandemic context of the study, more recent literature on health infodemics (2021-2024) should be added.

Adolescent psychosocial impact: ref. 35 (Landi et al., 2021) is the only reference addressing psychosocial impact in adolescents. Literature on coping in adolescents with chronic or medically uncertain illness should be added to contextualise the Discussion.

Several references appear incomplete in the manuscript. All references must be complete and formatted according to PLOS ONE style.

**********

what does this mean?). If published, this will include your full peer review and any attached files.

If you choose “no”, your identity will remain anonymous but your review may still be made public.

**Do you want your identity to be public for this peer review?**  For information about this choice, including consent withdrawal, please see our Privacy Policy

Reviewer #1: No

Reviewer #2: No

**********

[NOTE: If reviewer comments were submitted as an attachment file, they will be attached to this email and accessible via the submission site. Please log into your account, locate the manuscript record, and check for the action link "View Attachments". If this link does not appear, there are no attachment files.]

To ensure your figures meet our technical requirements, please review our figure guidelines: https://journals.plos.org/plosone/s/figures

You may also use PLOS’s free figure tool, NAAS, to help you prepare publication quality figures: https://journals.plos.org/plosone/s/figures#loc-tools-for-figure-preparation.

NAAS will assess whether your figures meet our technical requirements by comparing each figure against our figure specifications.

# Author comment: Author response to Decision Letter 1

**DOI:** 10.1371/journal.pone.0357185.r002

29 May 2026

PONE-D-25-58146

Perceptions, social representations, and experiences of suspected Lyme borreliosis among adolescents and their parents in France: a multicenter study.

PLOS One

Paris, April 16th, 2026

Dear Editor, Dear Reviewers,

Thank you for your careful reviews that helped improve our manuscript. Please find our answers below.

We hope they will meet your expectations.

Best regards,

The authors

Editor :

Provide intermediate analytic scaffolding showing how 809 codes were consolidated into three themes. This may include a codebook excerpt or table of subthemes, and add frequency language ("most," "several," "a minority") with approximate counts to characterize pattern distribution.

Thank you for your remark. We added a table of subthemes and the frequency of adverbs.

Both reviewers questioned the decision regarding pre-test family inclusion. Clearly state in the Results whether these interviews contributed substantively to final themes, whether guide changes occurred afterward, and whether sensitivity analyses were conducted.

Thank you for your remark. The pre-interviews provided additional insights for each theme and helped refine the subthemes. For example, as no fathers were included in the main interviews, it seemed valuable to retain the pre-interviews conducted with two fathers, particularly because they raised questions about the diagnosis of Lyme borreliosis and related care pathways. It also enabled to get a hole family (father + mother + 2 children). The pre-interviews contributed additional codes. The interview guide did not change substantially after the pre-interviews, so they were completed as well as the interviews. However, these preliminary discussions helped reinforce its validity. We added a paragraph to detail these points.

The reliance on a single reference (Laplantine) for "social representations" is insufficient given its centrality to your study. Engage with foundational social representations literature and more recent illness representation scholarship.

Thank you for your remark. We added references from Moscovici and Jodelet.

Expand discussion of how your affiliation with the TBD Reference Centre may have influenced data collection and interpretation, per COREQ requirements.

Thank you for your remark. We expanded the discussion with the following considerations.

SC conducted the interviews and had no prior contact with the patients in routine care, as she is a researcher in medical anthropology. She was recruited by the reference center to conduct this study because of her independence from patient care. All interviews were anonymized by SC before being independently coded by GP. Nevertheless, as some of the authors are physicians with expertise in the management of tick-borne diseases, including Lyme borreliosis, the interpretation of the data may have been influenced by their clinical experience. Therefore, a latent analytical approach was adopted to better understand participants’ perspectives in a comprehensive manner and to highlight practical implications for healthcare relationships. We also explicitly identified our own interpretive positions to support a critical analysis of the data, including reflexivity regarding our subjectivity. The perspectives were compared to identify convergences and divergences, leading to a final consensus.

Ensure all participant quotations are complete, clearly translated, and properly attributed. Truncated excerpts compromise assessment of interpretive rigor.

Thank you for your remark. Quotations were completed.

Specify participant contribution, whether responses came from adolescents, parents, or both, and consider presenting top terms in tabular format rather than relying solely on the word cloud.

Thank you for your remark. Quotations were completed with the participant contribution such as (A1) for adolescent 1, or (P1) for parent 1 for example.

Moreover, we prepared a table presenting top terms and their frequency (supplementary file 2B). The word cloud was kept in the supplementary files (supplementary file 2A).

The manuscript requires thorough editing by a native English speaker to address errors affecting readability.

Thank you for your remark. The manuscript was edited by a native English speaker.

Also, please consider including more recent literature on health infodemics (2021–2024) and adolescent coping with chronic/uncertain illness and correct incomplete references per PLOS ONE style.

Thank you for your remark. We corrected references according to PLOS ONE style. We also added the more recent references from 2025.

Journal Requirements:

When submitting your revision, we need you to address these additional requirements.

1. Please ensure that your manuscript meets PLOS ONE's style requirements, including those for file naming. The PLOS ONE style templates can be found at

https://journals.plos.org/plosone/s/file?id=wjVg/PLOSOne_formatting_sample_main_body.pdf and

https://journals.plos.org/plosone/s/file?id=ba62/PLOSOne_formatting_sample_title_authors_affiliations.pdf

2. In this instance it seems there may be acceptable restrictions in place that prevent the public sharing of your minimal data. However, in line with our goal of ensuring long-term data availability to all interested researchers, PLOS’ Data Policy states that authors cannot be the sole named individuals responsible for ensuring data access (http://journals.plos.org/plosone/s/data-availability#loc-acceptable-data-sharing-methods).

Data requests to a non-author institutional point of contact, such as a data access or ethics committee, helps guarantee long term stability and availability of data. Providing interested researchers with a durable point of contact ensures data will be accessible even if an author changes email addresses, institutions, or becomes unavailable to answer requests.

Before we proceed with your manuscript, please also provide non-author contact information (phone/email/hyperlink) for a data access committee, ethics committee, or other institutional body to which data requests may be sent. If no institutional body is available to respond to requests for your minimal data, please consider if there any institutional representatives who did not collaborate in the study, and are not listed as authors on the manuscript, who would be able to hold the data and respond to external requests for data access? If so, please provide their contact information (i.e., email address). Please also provide details on how you will ensure persistent or long-term data storage and availability.

3. Your ethics statement should only appear in the Methods section of your manuscript. If your ethics statement is written in any section besides the Methods, please delete it from any other section.

4. Please include captions for your Supporting Information files at the end of your manuscript, and update any in-text citations to match accordingly. Please see our Supporting Information guidelines for more information: http://journals.plos.org/plosone/s/supporting-information.

5. If the reviewer comments include a recommendation to cite specific previously published works, please review and evaluate these publications to determine whether they are relevant and should be cited. There is no requirement to cite these works unless the editor has indicated otherwise.

Reviewer 1

Reviewer #1:

1- The Results section reads more like an extended summary than an analytic presentation of grounded-theory outputs. Beyond stating “809 codes” and three themes, the manuscript does not show the intermediate analytic architecture that would allow a reader to judge adequacy and coherence, such as a concise codebook excerpt, illustrative subthemes, or a brief description of how the 809 codes were consolidated into the three themes. Without that scaffolding, the reader cannot assess whether the thematic structure is saturated, internally consistent, or selectively reported.

Thank you for your remark. We added a table of codes/subthemes /themes and the frequency of adverbs. We also added a table of the most frequent words, excluding adverbs.

2- Participant description is thin for a study that explicitly aims to examine perceptions and experiences across a potentially heterogeneous group. Table 1 is helpful as a first pass, but the Results should explicitly quantify key variation variables that were part of the sampling strategy, such as suspected versus confirmed Lyme borreliosis, symptom duration shorter than six months versus longer than six months, and rural versus urban exposure context, rather than leaving these to inference from a small table. This is particularly important given the modest number of adolescents and the risk that a few narratives dominate the synthesis.

Thank you for your remark. These key variable variables for sampling strategy were detailed in the methods’ section. We recalled them in the results’ section.

3- The inclusion of three pretest families within the analytic dataset needs a more explicit account in the Results, not only in the Methods. If those interviews were retained, it is important to state whether they contributed substantively to the final themes, whether any interview-guide changes occurred after them, and whether a sensitivity check was performed to ensure they did not disproportionately shape early coding trajectories. The current Results do not reassure the reader on this point, especially since saturation is claimed with a small sample.

Thank you for your remark. The pre-interviews provided additional insights for each theme and helped refine the subthemes. For example, as no fathers were included in the main interviews, it seemed valuable to retain the pre-interviews conducted with two fathers, particularly because they raised questions about the diagnosis of Lyme borreliosis and related care pathways. It also enabled to include a whole family (father, mother and 2 children). The pre-interviews contributed (xx) additional codes. The interview guide did not change substantially after the pre-interviews; however, these preliminary discussions helped reinforce its validity.

4- The Results would benefit from sharper within-theme contrasts between adolescents and parents, supported by a consistent strategy for quotation selection. The text asserts divergent experiences, such as parents describing Lyme borreliosis as “incurable” and adolescents expressing relative disinterest, but it remains unclear whether these are dominant patterns, minority voices, or evenly distributed across families. Consider adding brief frequency language that is standard in qualitative reporting, such as “most,” “several,” “a minority,” backed by counts or at least the number of families in which each pattern appeared.

Thank you for your remark that enables to highlight this important point of our study. We added a table with each theme and subthemes, and how they articulate between adolescents and parents.

5- The word-cloud result is presented as though it were an empirical “finding,” with “pain” said to be the most frequent word, yet the Results do not specify how many participants contributed to this prompt, whether responses were elicited from adolescents, parents, or both, and whether the prompt was asked once or across the three interviews per family. Given that the Discussion later interprets “pain” as a striking contrast, the Results should be strengthened with clear reporting of the underlying responses, ideally with a short table of the top terms and counts rather than relying on a word cloud as the primary vehicle.

Thank you for your remark. We prepared a table presenting top terms and their frequency. The word cloud was kept in the supplementary files.

6- Theme 2 is an important contribution, especially the tension between pediatric reference centers and alternative care pathways, and the destabilizing effect of inconsistent medical encounters. However, the Results blend together multiple phenomena, including discontinuation of follow-up, diagnosis contestation, and intrafamilial conflict, without distinguishing which are central versus peripheral. A clearer substructure inside Theme 2, with explicit subthemes and boundaries, would greatly improve interpretability and would reduce the impression that disparate observations have been grouped under an umbrella label.

Thank you for your remark. We added clearer subthemes in the table and added which families and how they were sharing each phenoma.

7- Claims of data saturation and thematic completeness would be more convincing if the Results included concrete indicators: when the third theme first appeared, whether later interviews added only nuance, and how the dyadic third interview influenced or challenged the individual interviews. Since the design involved three interviews per family, the Results should also clarify what unique analytic value came from the dyadic interview, rather than treating the dataset as if it were a single interview per participant.

Thank you for your remark. The third theme appeared at the first interview and was then further explored with different points of view through the 19 interviews. No new codes appeared after the 18th interview.

In line with grounded theory methodology, data collection and analysis were conducted iteratively, with constant comparison guiding the development of categories. While theoretical saturation was sought, it was considered in pragmatic terms. Data collection was stopped when categories were sufficiently developed in relation to the study aims and when additional data no longer contributed substantially new insights. This approach is consistent with the notion of “theoretical sufficiency,” acknowledging that full saturation may be difficult to achieve in practice. We assume that maybe we could have found more codes, especially as this issue is poorly studied (very few studies with adolescents and Lyme borreliosis).

A dyadic qualitative approach was adopted as the phenomenon under study was inherently relational and could not be fully understood through individual perspectives alone. This design enabled the exploration of how experiences, meanings, and decisions were co-constructed through interaction between participants. By conducting joint interviews, we were able to capture relational dynamics in situ, including agreements, disagreements, and negotiation processes, as well as asymmetries in knowledge and positioning.

For example, we identified new subthemes such as subtheme 1.3 in the table.

The dyadic approach also facilitated the identification of convergent and divergent perspectives within the same relational context, providing a more nuanced understanding of participants’ experiences. This was particularly relevant in the context of Lyme borreliosis, where diagnostic processes and care pathways are often shaped by interactions between patients, their relatives, and healthcare professionals.

Reviewer 2

Reviewer #2: REVIEW REPORT

Manuscript ID: PONE-D-25-58146

Article Type: Research Article

Title: Perceptions, social representations, and experiences of suspected Lyme borreliosis among adolescents and their parents in France: a multicenter study.

Summary: This manuscript presents an original exploratory qualitative study examining how adolescents and their parents perceive, represent, and experience Lyme borreliosis (LB) in the context of a suspected or confirmed diagnosis. It reports data collected through semi-structured interviews analysed using grounded theory with a dyadic approach. The study was conducted in France, in two university hospitals affiliated with a national Tick-Borne Disease (TBD) Reference Centre. Eight families (19 individuals) participated. The title is informative and accurately reflects the study content.

General comments

This study represents a contribution to the literature on LB in paediatrics. The available literature focused on the social representations and lived experiences of adolescents with suspected LB and their parents is scarce. The dyadic methodology is a methodological strength that sets this work apart from single-respondent designs. The grounded theory approach is well-matched to the exploratory research question.

The manuscript is, however, hampered by language issues and sever

Attachment Submitted filename: Response to Reviewers.docx

# Peer review: Decision Letter 1

**DOI:** 10.1371/journal.pone.0357185.r003

**Authors:** Ernest Tambo

13 Aug 2026

Perceptions, social representations, and experiences of suspected Lyme borreliosis among adolescents and their parents in France: a multicenter study.

PONE-D-25-58146R1

Dear Dr. ,

We’re pleased to inform you that your manuscript has been judged scientifically suitable for publication and will be formally accepted for publication once it meets all outstanding technical requirements.

Within one week, you’ll receive an e-mail detailing the required amendments. When these have been addressed, you’ll receive a formal acceptance letter and your manuscript will be scheduled for publication.

An invoice will be generated when your article is formally accepted. Please note, if your institution has a publishing partnership with PLOS and your article meets the relevant criteria, all or part of your publication costs will be covered. Please make sure your user information is up-to-date by logging into Editorial Manager at Editorial Manager® and clicking the ‘Update My Information' link at the top of the page. For questions related to billing, please contact billing support.

If your institution or institutions have a press office, please notify them about your upcoming paper to help maximize its impact. If they’ll be preparing press materials, please inform our press team as soon as possible -- no later than 48 hours after receiving the formal acceptance. Your manuscript will remain under strict press embargo until 2 pm Eastern Time on the date of publication. For more information, please contact onepress@plos.org.

Kind regards,

Ernest Tambo

Academic Editor

PLOS One

Additional Editor Comments (optional):

Reviewers' comments:

Reviewer's Responses to Questions

**Comments to the Author**

Reviewer #1: (No Response)

Reviewer #2: All comments have been addressed

**********

2. Is the manuscript technically sound, and do the data support the conclusions?

Reviewer #1: (No Response)

Reviewer #2: Yes

**********

3. Has the statistical analysis been performed appropriately and rigorously?

Reviewer #1: (No Response)

Reviewer #2: Yes

**********

4. Have the authors made all data underlying the findings in their manuscript fully available?

The PLOS Data policy

Reviewer #1: (No Response)

Reviewer #2: Yes

**********

5. Is the manuscript presented in an intelligible fashion and written in standard English?

Reviewer #1: (No Response)

Reviewer #2: Yes

**********

Reviewer #1: After thoroughly reviewing the revised manuscript and the authors' detailed responses to the initial comments, the improvements made are satisfactory.

Reviewer #2: The authors have addressed the points raised in my review. I therefore consider that the manuscript now meets the conditions for publication.

**********

what does this mean?). If published, this will include your full peer review and any attached files.

If you choose “no”, your identity will remain anonymous but your review may still be made public.

**Do you want your identity to be public for this peer review?**  For information about this choice, including consent withdrawal, please see our Privacy Policy

Reviewer #1: No

Reviewer #2: **Yes:** Sofia Ferreira Anastácio

**********

# Editor report: Acceptance letter

**DOI:** 10.1371/journal.pone.0357185.r004

**Authors:** Ernest Tambo

PONE-D-25-58146R1

PLOS One

Dear Dr. Raffetin,

I'm pleased to inform you that your manuscript has been deemed suitable for publication in PLOS One. Congratulations! Your manuscript is now being handed over to our production team.

At this stage, our production department will prepare your paper for publication. This includes ensuring the following:

* All references, tables, and figures are properly cited

* All relevant supporting information is included in the manuscript submission,

* There are no issues that prevent the paper from being properly typeset

You will receive further instructions from the production team, including instructions on how to review your proof when it is ready. Please keep in mind that we are working through a large volume of accepted articles, so please give us a few days to review your paper and let you know the next and final steps.

Lastly, if your institution or institutions have a press office, please let them know about your upcoming paper now to help maximize its impact. If they'll be preparing press materials, please inform our press team within the next 48 hours. Your manuscript will remain under strict press embargo until 2 pm Eastern Time on the date of publication. For more information, please contact onepress@plos.org.

You will receive an invoice from PLOS for your publication fee after your manuscript has reached the completed accept phase. If you receive an email requesting payment before acceptance or for any other service, this may be a phishing scheme. Learn how to identify phishing emails and protect your accounts at https://explore.plos.org/phishing.

If we can help with anything else, please email us at customercare@plos.org.

Thank you for submitting your work to PLOS One and supporting open access.

Kind regards,

PLOS One Editorial Office Staff

on behalf of

Dr. Ernest Tambo

Academic Editor

PLOS One
